# Emergence of New Delhi Metallo-β-Lactamase (NDM-5) in *Klebsiella quasipneumoniae* from Neonates in a Nigerian Hospital

**DOI:** 10.1128/mSphere.00685-18

**Published:** 2019-03-13

**Authors:** Lauren M. Brinkac, Richard White, Roshan D’Souza, Kevin Nguyen, Stephen K. Obaro, Derrick E. Fouts

**Affiliations:** a J. Craig Venter Institute, Rockville, Maryland, USA; b University of Nebraska Medical Center, Omaha, Nebraska, USA; c Department of Pediatrics, University of Abuja Teaching Hospital, Abuja, Nigeria; d International Foundation against Infectious Diseases in Nigeria (IFAIN), Abuja, Nigeria; Antimicrobial Development Specialists, LLC

**Keywords:** ESBL, *Enterobacteriaceae*, *Klebsiella*, New Delhi metallo-β-lactamase (NDM-5), Nigeria, antimicrobial resistance, bacteria, carbapenemase, neonate

## Abstract

Outbreaks of infection occur more often than they are reported in most developing countries, largely due to poor diagnostic services. A *Klebsiella* species bacteremia outbreak in a newborn unit with high mortality was recently encountered at a location being surveilled for childhood bacteremia. These surveillance efforts offered the opportunity to determine the cause of this neonatal outbreak. In this report, we present the whole-genome sequences of New Delhi metallo-β-lactamase (NDM-5)-containing Klebsiella quasipneumoniae subsp. *similipneumoniae* bloodstream isolates from a neonatal bacteremia outbreak at a tertiary hospital in Nigeria and as part of the largest collection of K. pneumoniae bloodstream isolates from children in Africa. Comparative analysis of the genetic environment surrounding the NDM-5 genes revealed nearly perfect sequence identity to *bla*_NDM-5_-bearing IncX3-type plasmids from other members of the *Enterobacteriaceae*.

**IMPORTANCE** Carbapenem-resistant Klebsiella pneumoniae is of global health importance, yet there is a paucity of genome-based studies in Africa. Here we report fatal blood-borne NDM-5-producing K. quasipneumoniae subsp. *similipneumoniae* infections from Nigeria, Africa. New Delhi metallo-β-lactamase (NDM)-producing *Klebsiella* spp. are responsible for high mortality and morbidity, with the NDM-5 variant showing elevated carbapenem resistance. The prevalence of NDM-5 in *Klebsiella* has been limited primarily to K. pneumoniae, with only one isolate being collected from Africa. During an outbreak of sepsis in a teaching hospital in Nigeria, five NDM-5-producing K. quasipneumoniae subsp. *similipneumoniae* sequence type 476 isolates were identified. Given the increased resistance profile of these strains, this study highlights the emerging threat of *bla*_NDM-5_ dissemination in hospital environments. The observation of these NDM-5-producing isolates in Africa stresses the urgency to improve monitoring and clinical practices to reduce or prevent the further spread of resistance.

## Abstract (precis)

Carbapenem-resistant Klebsiella pneumoniae is of global health importance, yet there is a paucity of genome-based studies in Africa. Here we report fatal blood-borne NDM-5-producing K. quasipneumoniae subsp. *similipneumoniae* infections from Nigeria, Africa. New Delhi metallo-β-lactamase (NDM)-producing *Klebsiella* spp. are responsible for high mortality and morbidity, with the NDM-5 variant showing elevated carbapenem resistance. The prevalence of NDM-5 in *Klebsiella* has been limited primarily to K. pneumoniae, with only one isolate being collected from Africa. During an outbreak of sepsis in a teaching hospital in Nigeria, five NDM-5-producing K. quasipneumoniae subsp. *similipneumoniae* sequence type 476 isolates were identified. Given the increased resistance profile of these strains, this study highlights the emerging threat of *bla*_NDM-5_ dissemination in hospital environments. The observation of these NDM-5-producing isolates in Africa stresses the urgency to improve monitoring and clinical practices to reduce or prevent the further spread of resistance.

## INTRODUCTION

The global dissemination of carbapenemase-producing *Klebsiella* spp. poses a serious public health threat. Among the newly emerging carbapenemases, NDM is one of the most clinically significant due to its increased resistance phenotype, rapid and ongoing evolution, and global dissemination. Since NDM-1 was initially identified from Klebsiella pneumoniae in 2008 (1), 16 new *bla*_NDM_ alleles have been identified, with most of them originating from Asia. NDM producers now include multiple bacterial genera and have spread to virtually every continent (2), largely due to the plasmid-mediated transfer of *bla*_NDM_.

In 2011, a multidrug-resistant (MDR) Escherichia coli strain isolated in the United Kingdom from a patient returning from a recent hospitalization in India was found to harbor NDM-5 (3). In comparison to the prevalent NDM-1 allele, NDM-5, a 2-amino-acid variant, conferred elevated carbapenem resistance (3) and has subsequently been identified in isolates from other members of the *Enterobacteriaceae* family worldwide (4–30). Among the *Enterobacteriaceae*, reports of NDM-5-producing *Klebsiella* spp. are sporadic (17–19, 21–24, 31), with only one isolate collected from a hospitalized infant in northern Africa (20). Furthermore, the prevalence of NDM-5 in *Klebsiella* has been limited primarily to K. pneumoniae strains.

In this study, we report on an NDM-5-producing K. quasipneumoniae subsp. *similipneumoniae* strain representing the sequence type 476 (ST476) clonal group isolated from neonates at the University of Abuja Teaching Hospital, Gwagwalada, Nigeria.

## RESULTS AND DISCUSSION

### Clinical setting of *Klebsiella* outbreak.

The evaluation of the causative agent of this outbreak was facilitated by one of the surveillance laboratories for the Community-Acquired Bacteremic Syndrome in Young Nigerian Children (CABSYNC) program, which is located at the University of Abuja Teaching Hospital, Gwagwalada, Nigeria, and the diagnostic service was offered at no cost to the parents of these babies. At most health care facilities in Nigeria and, indeed, throughout sub-Saharan Africa, diagnostic microbiology laboratories are not readily available, and where they are available, the service is neither free nor affordable; thus, most septic newborns are treated empirically.

An outbreak of neonatal sepsis occurred during the month of April 2016, when there was a high admission in the special care baby unit of the University of Abuja Teaching Hospital, Gwagwalada, in central Nigeria. This is one of two special care neonatal units in the Federal Capital Territory, both of which cater to a population of over 3 million (National Bureau of Statistics, Nigeria). The bed occupancy rates typically exceed the total number of beds, with babies being nursed on Resuscitaire units (Dräger, Lübeck, Germany) and less critically ill babies occasionally sharing cots. Mechanized respiratory support was limited to continuous positive airway pressure (CPAP). The outbreak prompted an increased level of infection control, such as enforced hand washing, restricted access to the unit, and temporary closing of the unit for 4 days for sanitization, which presumably led to resolution of the outbreak by the beginning of May 2016.

### Sequencing of neonatal bloodstream isolates of MDR *Klebsiella* spp.

Illumina NextSeq genome sequencing was performed on seven bloodstream isolates of *Klebsiella* spp. from babies being treated in the University of Abuja Teaching Hospital, Gwagwalada, in central Nigeria, as part of an ongoing (2012 to 2016) surveillance for community-acquired bacteremic syndromes (CABSYNC). Part of this collection included five isolates obtained during the April 2016 outbreak of neonatal sepsis. The resulting *de novo* assembly Illumina sequence coverage of five of the seven isolates was between 90-fold (for isolate G4612) and 230-fold (for isolate G4584) across an average of 121 contigs per genome (minimum, 46 for G4704; maximum, 146 for G4612), resulting in average draft genome sizes of between 5.4 Mbp (G4704) and 5.8 Mbp (G4601) (Table 1). Two representative isolates, G4584 and G747, which received additional Oxford Nanopore minION sequencing, resulted in a hybrid *de novo* assembly of seven circular contigs including one chromosome (5,446,060 bp) and six plasmids (3,733 bp to 218,935 bp) and a *de novo* assembly of six circular contigs including one chromosome (5,395,457 bp) and five plasmids (3,733 bp to 218,944 bp), respectively. The resulting hybrid *de novo* assembly sequence coverage of G4584 was 760-fold (234 times by Illumina NextSeq sequencing and 525 times by Oxford Nanopore sequencing), and that of G747 was 164-fold (105 times by Illumina NextSeq sequencing and 60 times by Oxford Nanopore sequencing).

**TABLE 1:** Select genomic features and metadata for the K. quasipneumoniae subsp. *similipneumoniae* genomes sequenced in this study^*a*^

BioSample accession no.	Strain	Length (Mbp)	*N*_50_	MLST ST	MLST allelic profile^*b*^	Patient age (days)	Date of isolation	Presence of NDM
SAMN05960914	G747	5.9	5395457	476	18-22-26-22-93-37-99	6	25 Feb 2013	−
SAMN05960931	G4582	5.8	219823	476	18-22-26-22-93-37-99	2	14 Apr 2016	+
SAMN05960932	G4584	5.9	5446060	476	18-22-26-22-93-37-99	1	15 Apr 2016	+
SAMN05960934	G4593	5.8	226966	476	18-22-26-22-93-37-99	1	20 Apr 2016	+
SAMN05960936	G4601	5.8	208789	476	18-22-26-22-93-37-99	1	26 Apr 2016	+
SAMN05960939	G4612	5.8	208978	476	18-22-26-22-93-37-99	4	04 May 2016	+
SAMN05960940	G4704	5.4	368354	1031	18-22-18-23-134-13-51	2	18 Jul 2016	−

a All isolates originated from the blood of patients at the University of Abuja Teaching Hospital, Gwagwalada, Nigeria.

b The alleles are for *gapA****-****infB****-****mdh****-****pgi****-****phoE****-****rpoB****-****tonB*.

### Taxonomic classification.

**FIG 1:**
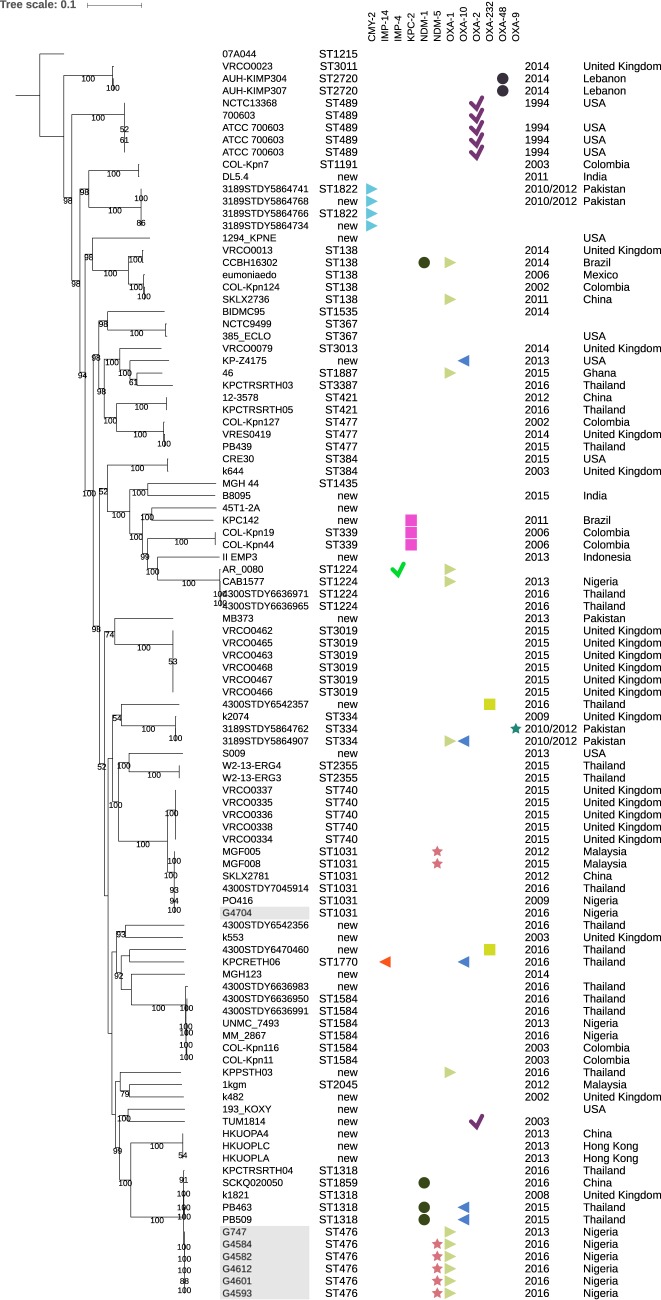
Phylogenetic analysis of K. quasipneumoniae subsp. *similipneumoniae* isolates. The resulting tree was rooted with type strain K. quasipneumoniae subsp. *similipneumoniae* 07A044 (GenBank accession no. NZ_CBZR010000000) and annotated with genotypes of resistance to common carbapenemases. The isolates discussed in this study are highlighted. The predicted sequence type (ST), isolation year, and geographical origin are displayed. Novel STs are indicated by “new.” The numbers at the nodes represent >50% bootstrap support. The scale bar represents the number of nucleotide substitutions per site.

Phylogenetic characterization via *in silico* multilocus sequence typing (MLST) and determination of single nucleotide polymorphisms (SNPs) of publicly available *Klebsiella* species genome sequences (*n* = 4,963), including *Klebsiella* species isolates from Nigeria (*n* = 93), revealed that the Nigerian isolates in this study could be taxonomically classified as K. quasipneumoniae subsp. *similipneumoniae* (Fig. 1). The taxonomy of all *Klebsiella* species genomes was confirmed by the average nucleotide sequence identity (ANI). Those phylogenetically characterized as K. quasipneumoniae subsp. *similipneumoniae* (*n* = 102) in this study had >98% ANI to K. quasipneumoniae subsp. *similipneumoniae* 07A044^T^ (see Table S1 in the supplemental material). Sequence type 476 (ST476) was identified in six of the K. quasipneumoniae subsp. *similipneumoniae* isolates collected from the University of Abuja Teaching Hospital, Gwagwalada, Nigeria (Table 1), representing a clonal group with 98.94% to 100% identity by pairwise ANI.

10.1128/mSphere.00685-18.1 TABLE S1 Klebsiella quasipneumoniae subsp. *similipneumoniae* genomes analyzed as part of this study. Download Table S1, PDF file, 0.08 MB. Copyright © 2019 Brinkac et al. 2019 Brinkac et al. This content is distributed under the terms of the Creative Commons Attribution 4.0 International license.

### Phenotypic susceptibility characterization.

Antimicrobial susceptibility testing of the K. quasipneumoniae subsp. *similipneumoniae* isolates demonstrated various degrees of resistance to broad-spectrum antibiotics (Table 2). The April 2016 outbreak isolates, G4582, G4584, G4593, G4601, and G4612, were resistant to virtually every antibiotic tested, including the carbapenems imipenem, meropenem, and ertapenem (MICs, >32 μg/ml). Exceptions for resistance to other antibiotics included intermediate resistance to amikacin (MICs, 24 to 32 μg/ml) and, in the case of G4612, also resistance to chloramphenicol (MIC, 16 μg/ml). No other K. quasipneumoniae subsp. *similipneumoniae* isolate obtained as part this study had a high level of resistance to all antibiotics tested, including the carbapenems. G747, which shares the same sequence type, ST476, and five of six plasmids with G4584 but was isolated from the same hospital 3 years earlier, exhibited a similar resistance profile but was sensitive to imipenem (MIC, 0.5 μg/ml), meropenem (MIC, 0.125 μg/ml), and ertapenem (MIC, 0.064 μg/ml), as well as cefoxitin (MIC, 8 μg/ml). Similarly, G4704, obtained 3 months later, was also sensitive to carbapenems (imipenem, meropenem, and ertapenem), as well as cefoxitin, ceftazidime, cefepime, piperacillin-tazobactam, and amikacin, and showed intermediate resistance to amoxicillin-clavulanic acid.

**TABLE 2:** Antimicrobial susceptibility testing of K. quasipneumoniae subsp. *similipneumoniae* isolates

Strain	MIC (μg/ml)^*a*^																				
	AMP	AMC	CEF	FOX	CAZ	CRO	CTX	FEP	TZP	IPM	MEM	ETP	GEN	AMK	TOB	SXT	TET	CIP	CHL	ESBL CAZ-CLA	ESBL CTX-CLA
G747	>256	48	>256	8	>256	>256	>256	>256	>256	0.5	0.125	0.064	256	24	48	>32	>256	32	12	>32/0.38 (+)	>16/0.19 (+)
G4582	>256	>256	>256	>256	>256	>256	>256	>256	>256	>32	>32	>32	>256	24	256	>32	>256	>32	24	>32/>4 (−)	>16/>1 (−)
G4584	>256	>256	>256	>256	>256	>256	>256	>256	>256	>32	>32	>32	>256	32	192	>32	>256	>32	24	>32/>4 (−)	>16/>1 (−)
G4593	>256	256	>256	>256	>256	>256	>256	>256	>256	>32	>32	>32	>256	24	192	>32	>256	32	24	>32/4 (+)	>32/0.19 (+)
G4601	>256	256	>256	>256	>256	>256	>256	>256	>256	>32	>32	>32	256	32	64	>32	>256	>32	32	>32/>4 (−)	>16/>1
G4612	>256	>256	>256	>256	>256	>256	>256	>256	>256	>32	>32	>32	>256	32	256	>32	>256	>32	16	>32/>4 (−)	>16/>1 (−)
G4704	>256	16	>256	8	1.5	>256	>256	6	6	0.5	0.094	0.023	>256	4	12	>32	>256	4	>256	<0.5/0.25 (−)	>16/0.125 (+)

a AMP, ampicillin; AMC, amoxicillin-clavulanic acid; CEF, cephalothin; FOX, cefoxitin; CAZ, ceftazidime; CRO, ceftriaxone; CTX, cefotaxime; FEP, cefepime; TZP, piperacillin-tazobactam; IPM, imipenem; MEM, meropenem; ETP, ertapenem; GEN, gentamicin; AMK, amikacin; TOB, tobramycin; SXT, trimethoprim-sulfamethoxazole; TET, tetracycline; CIP, ciprofloxacin; CHL, chloramphenicol; CAZ-CLA, ESBL ceftazidime-clavulanic acid; CTX-CLA, ESBL cefotaxime-clavulanic acid; +, positive; −, negative.

### Genotypic characterization of NDM-5-containing K. quasipneumoniae subsp. *similipneumoniae* isolates.

Multidrug-resistant K. quasipneumoniae subsp. *similipneumoniae* isolates G4582, G4584, G4593, G4601, and G4612 were found to harbor β-lactamase genes *bla*_CTX-M-15_, *bla*_NDM-5_, *bla*_OKP-B-6_, *bla*_OXA-1_, and *bla*_TEM-1_, and non-β-lactam acquired resistance genes included *aac(6')-Ib-cr*, *ble*_MBL_, *qnrB1*, and *sul2* (Table S2). Complete genomic sequencing of G4584 revealed a circularized IncX3-type plasmid carrying the NDM-5 allele in the outbreak strain and confirmed the absence of the *bla*_NDM-5_-bearing plasmid in the preoutbreak strain, G747. Compared with other publicly available K. quasipneumoniae subsp. *similipneumoniae* strains (*n* = 102), this is the first report of NDM-5 in an ST476 isolate (Fig. 1; Table S1), and to the best of our knowledge, this is the first occurrence of an NDM-5-producing *Klebsiella* sp. in Nigeria and one of few *Klebsiella* species NDM producers in Africa (20, 32–40).

10.1128/mSphere.00685-18.2 TABLE S2 *In silico*-predicted antimicrobial resistance genes. Values represent the percent identity of the identified gene in each isolate to a CARD AMR gene. Download Table S2, PDF file, 0.04 MB. Copyright © 2019 Brinkac et al. 2019 Brinkac et al. This content is distributed under the terms of the Creative Commons Attribution 4.0 International license.

Screening for putative virulence genes revealed no difference between the carriage of a virulence-associated gene/gene clusters among NDM-5-containing and NDM-5-noncontaining Nigerian isolates. Genes coding for the urease (*ureABCDEFG*) and fimbria (*mrkABCDFHIJ* and *fimFGH*) gene clusters, glucuronic acid transferase (*wabG*), the siderophores enterobactin (*entABCDEF*), and ferric iron uptake (*kfuABC*) were detected in all seven Nigerian isolates, while genes coding for the allantoinase gene cluster (*allABCDRS*), the two-component system KvgAS (*kvgAS*), and mucoid phenotype regulators (*rmpA* and *rmpA2*) and the *glxKR*, *ybbWY*, *ylbEF*, *hyi*, *arcC*, and *fdrA* virulence-associated genes were absent.

### Comparative analysis of the NDM-5 genetic environment.

**FIG 2:**
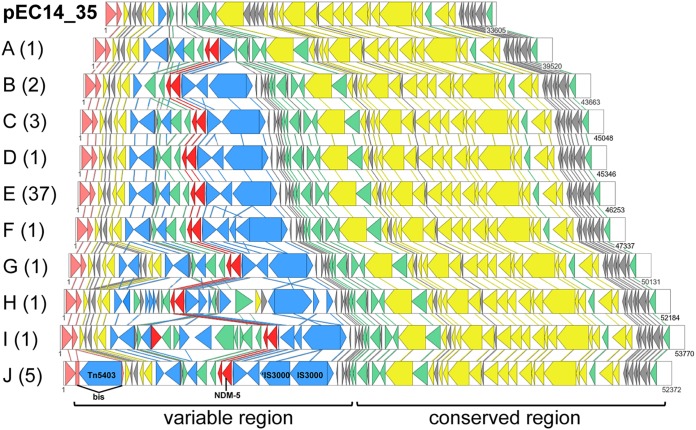
Linear comparison of the genetic environment of the *bla*_NDM-5_ region found on IncX3 plasmids from members of the *Enterobacteriaceae*. E. coli plasmid pEC14_35 (GenBank accession no. JN935899) represents the prototypical IncX3 plasmid that lacks known antibiotic resistance determinants. Group A represents the sequence from E. coli plasmid pNDM5-NJ-IncX3 (GenBank accession no. KX447767). Group B consists of two sequences (GenBank accession no. NEWC01000014.1 and NEWB01000014.1), both from K. quasipneumoniae. Group C has 3 members: sequences with GenBank accession no. CP027204 and MF547511 from E. coli and the sequence with GenBank accession no. CP028536 from Enterobacter hormaechei. Group D represents the sequence with GenBank accession no. KY041843 from E. coli plasmid pZHDC40. Group E contains 37 examples consisting of sequences with GenBank accession no. CP019073, CP021692, CP021738, CP024825, CP025948, CP026577, CP028577, CP028705, CP029245, KF220657, KT824791, KU167608, KU167609, KX023261, KX507346, KX960109, MF547507, MF547508, MF547509, MF547510, MF679143, MG252891, MG545911, MG825368, MG825382, MG825384, and MH094148 from E. coli; CM007781 and MTKV01000083 from Salmonella enterica subsp. *enterica* serovar Typhimurium; CP024820 from Citrobacter freundii; CP014006, KF220657, KU761328, MH161191, and MH341575 from K. pneumoniae; CP022351 and CP023188 from Klebsiella michiganensis; and MG833406 from Klebsiella oxytoca. Group F represents the sequence with GenBank accession no. KY435936 from E. coli plasmid pNDM5_WCHEC0215. Group G represents the sequence with GenBank accession no. MG591703 from E. coli plasmid pNDM-EC36. Group H represents the sequence with GenBank accession no. MH107030 from K. pneumoniae plasmid pTBCZNDM01. Group I represents the sequence with GenBank accession no. MH349095 from E. coli plasmid pMTC948. Group J contains 5 examples (from this study), sequences with GenBank accession no. NZ_NFXE01000097 (G4582), CP034133 (G4584), NZ_NFXD01000099 (G4593), NZ_NFXB01000102 (G4601), and NZ_NFWY01000105 (G4612), all from K. quasipneumoniae subsp. *similipneumoniae*. Arrows indicate protein-coding genes (CDSs) drawn to scale and colored as follows: salmon for factors involved in plasmid replication, yellow for factors involved in plasmid maintenance and mobility, red for antibiotic resistance determinants, blue for mobile elements, green for other known proteins, and gray for unknown proteins. Homologous CDSs between adjacent groups are joined vertically by colored lines.

IncX3-type plasmids have a narrow host range and are found primarily within the *Enterobacteriaceae* (41), and IncX3-type plasmids containing *bla*_NDM-5_ were found within several members of the *Enterobacteriaceae*, including E. coli, Salmonella enterica subsp. *enterica* serovar *Typhimurium*, K. pneumoniae, K. michiganensis, and K. quasipneumoniae (Fig. 2). Comparative analysis of all fully sequenced IncX3 plasmids containing an NDM-5 allele was performed to assess the genetic context of the NDM-5 gene. Ten different structural forms were identified from a total of 48 plasmid sequences available in GenBank and the five outbreak isolates from this study and are denoted groups A to J (Fig. 2). The plasmid backbone was nearly identical across the groups (conserved region, Fig. 2), with all plasmids carrying genes for replication (*pir* and *bis*), partitioning (*parA-parB*), entry exclusion (*eex*), maintenance (*topB* and *stpA*), and conjugative transfer (type IV secretion system and *taxA*, *taxB*, *taxC*, and *taxD*). However, there were some structural differences resulting from potential insertions/deletions of components of existing insertion sequence (IS) elements (Fig. 2, blue arrows). For example, group F may have had a second insertion of IS*5*, disrupting the IS*3000* transposase, and groups B, D to G, and J displayed apparent insertions of IS*Aba125* between IS*3000* and IS*5* that were lacking in the prototype sequences from group C. It is difficult to determine from the available sequence data whether the group C sequences resulted from deletion of these IS elements or whether the other groups represented novel IS insertions relative to group C. Only group I, represented by the unpublished E. coli plasmid pMTC948, possesses an additional *bla* gene (*bla*_SHV_). These results suggest that the variable region (Fig. 2) may be highly dynamic, but other than the loss of a promoter from the end of the IS*Aba125* fragment, previously shown to drive the expression of *bla*_NDM-1_ and *ble*_MBL_ (42), it is unclear if these differences have any effect on the expression of *bla*_NDM-5_, *ble*_MBL_, or the accessory genes *trpF* and *dsbC*. Both *trpF* and *dsbC* appeared to be tightly linked to *bla*_NDM-5_ in all sequences examined, with the exception of the sequence of the unpublished plasmid pTBCZNDM01 in group H, which lacked both *trpF* and *dsbC*, suggesting a critical role either in the stability, retention, or spread of this element or in facilitating enzyme functionality. The NDM-5-containing plasmid from K. quasipneumoniae subsp. *similipneumoniae* is structurally similar to members of group E, with two differences: a partial duplication of the IS*3000* element and truncation of the accessory replication protein Bis via insertion of the Tn*5403* transposon. Inactivation of *bis* results in the loss of beta origin replication, but not alpha or gamma origin replication, in the prototypical IncX family plasmid R6K (43). Given that similar IncX3 plasmids possess multiple origins of replication (44), it is unlikely that the loss of Bis will reduce the spread of antibiotic resistance.

### Transmission of NDM-5.

The transmission of a plasmid carrying NDM-5 was evidenced by performing conjugation experiments with donor cells harboring the NDM-5-containing plasmid. Due to the extensive drug resistance of strain G4584, the NDM-5-containing plasmid was electroporated into E. coli DH10B and subsequently transferred to a separate E. coli strain (JW2786-1). JW2786-1 cells harboring the NDM-5-containing plasmid grew on CHROMagar KPC plates supplemented with kanamycin only if the NDM-5-containing plasmid transferred via conjugation from DH10B to JW2786-1. Transfer of the *bla*_NDM-5_ IncX3-type plasmid to recipient cells (E. coli JW2786-1) was confirmed by PCR and the acquisition of carbapenem resistance in the recipient strain, which was measured using disk diffusion. The recipient strain turned resistant to meropenem (decrease in zone diameter, 35 mm to 15 mm), cefoxitin (23 mm to 6 mm), amoxicillin-clavulanic acid (22 mm to 8 mm), and cefepime (36 mm to 12 mm) after acquiring the *bla*_NDM-5_ IncX3-type plasmid.

In conclusion, we describe the occurrence of clonal (ST476) NDM-5-producing K. quasipneumoniae subsp. *similipneumoniae* isolates in Africa with an IncX3-type plasmid highly similar to the plasmids found in other members of the *Enterobacteriaceae*. The original source and transmission route of these isolates are unclear, but the close proximity of patients within the hospital when this outbreak occurred could have played a role in its transmission to other neonates in the unit. After the introduction of infection control measures, no isolates with NDM-5 were identified. Given the increased resistance profile of these strains and the associated high mortality rate among infected patients, this study highlights the emerging threat of the plasmid-mediated transfer and spread of *bla*_NDM-5_ in hospital environments. Furthermore, the increasing pervasiveness of NDM-5 enzymes confirmed in North Africa and the now newly identified occurrence in western Africa stress the urgency to improve monitoring and clinical practices to reduce or prevent further the spread of resistance.

## MATERIALS AND METHODS

### Participant description.

Children were enrolled per a previously published protocol (45, 46). Briefly, children less than 5 years old who presented to any of the enrolling clinical facilities in the Federal Capital Territory of Nigeria with clinical symptoms that were suggestive of bacteremia were enrolled following the provision of informed consent by the parent or guardian.

### Bacterial isolation and culturing.

Blood sampling and processing were as previously described (45, 46). Briefly, only aerobic blood culture bottles were utilized, and cultures were held in a Bactec 9050 incubator for a maximum of 5 days. Bacteria were identified by a combination of morphology and biochemical testing for *Enterobacteriaceae* using an API 20E system (bioMérieux, France). All blood-borne bacterial isolates that were recovered from September 2012 to September 2016 and that were identified as *Klebsiella* spp. were shipped to the University of Nebraska, where secondary confirmation of their identity was performed using standard biochemical tests. For genomic DNA isolation, *Klebsiella* isolates were cultured aerobically at 250 rpm in 1.5 ml brain heart infusion (BHI) medium overnight at 37°C. Only one bacterial isolate was processed per participant.

### AST.

Antibiotic susceptibility testing (AST) was performed by the University of Nebraska Medical Center (UNMC) using the Etest (bioMérieux, France). The antimicrobial drugs tested were ampicillin, amoxicillin-clavulanate, cephalothin, cefoxitin, ceftazidime, ceftriaxone, cefotaxime, cefepime, piperacillin-tazobactam, imipenem, meropenem, ertapenem, gentamicin, amikacin, tobramycin, trimethoprim-sulfamethoxazole, tetracycline, ciprofloxacin, chloramphenicol, the extended-spectrum β-lactamase (ESBL) ceftazidime-clavulanic acid, and the ESBL cefotaxime-clavulanic acid.

### DNA isolation and whole-genome sequencing.

Using a 1-ml overnight BHI culture, genomic DNA was isolated using a MasterPure Gram-positive DNA purification kit (Epicentre). The extracted genomic DNA was resuspended in ∼30 µl Tris-EDTA (TE) buffer and quantified using a NanoDrop spectrophotometer. Paired-end 150-bp Nextera XT libraries of whole genomic DNA were sequenced on an Illumina NextSeq sequencer with a target average coverage of 100-fold. All sequences were *de novo* assembled individually using the SPAdes algorithm (47). The genomes of strains G747 and G4584 were selected for additional sequencing using the Oxford Nanopore minION technology (one-dimensional sequencing on an R9.4 flow cell). G4584 was hybrid *de novo* assembled using reads from both the Illumina NextSeq and Oxford Nanopore minION sequencers with the Unicycler (v0.4.6) assembler (48). G747 was *de novo* assembled using the long-read assembler Canu (v1.7.1) (49), and the consensus sequence was generated using the Racon (v1.3.1) program (50). The circular nature of the assembled contigs was determined based on the presence of nearly identical repeats at the contig ends. The redundant regions were trimmed from one end, and the contig orientation and starting position were adjusted such that the first gene of the chromosome and plasmids was *dnaA* and *repA*, respectively. The final G747 assembly was polished using the Pilon (v1.22) tool (51) and the Illumina reads. All assembled sequences were annotated with NCBI’s prokaryotic genome annotation pipeline (PGAP) (52).

### Genomic analysis.

*In silico* MLST of the seven-locus K. pneumoniae Pasteur Institute MLST scheme (http://bigsdb.pasteur.fr/klebsiella/) and identification of virulence factors were performed using the LOCUST typer (53). Resistance Gene Identifier (RGI) software (54) in strict mode was used to predict the antibiotic resistome from whole-genome sequence data using the Comprehensive Antibiotic Resistance Database (CARD) (54–56). A whole-genome alignment was inferred from SNPs identified by the Northern Arizona SNP Pipeline (NASP; v1.0.2) (57) using the genome of K. quasipneumoniae subsp. *similipneumoniae* 07A044^T^ (GenBank accession no. NZ_CBZR010000000) as the reference. The resulting alignment was run through the Gubbins (v2.2.1) program (58) to filter out the effects of recombination on our maximum likelihood phylogenetic tree, generated using the RAxML tool (59) under the GTRCAT model with 100 bootstrap replicates. The resulting tree was rendered with metadata annotated using the Interactive Tree of Life (iTOL) (60–62). Taxonomic assignments were confirmed using the Mash (v1.1.1) program, which is based on ANI (63).

### Plasmid and NDM-5 synteny analysis.

Plasmid incompatibility groups were identified using Plasmid Finder (v1.3) software (https://cge.cbs.dtu.dk/services/PlasmidFinder/) (64). Coding sequences (CDSs) were determined based on the available NCBI PGAP annotations, and nucleotide comparisons of CDSs between fully sequenced IncX3-type plasmids containing an NDM-5 allele were performed using the NCBI legacy BLAST (v2.2.9) program. The presence of transposons and insertion sequences was confirmed using the IS Finder database (65). A linear illustration of the NDM-5-containing plasmids was generated using the SimpleSynteny tool (66) and edited using Adobe Illustrator software.

### Plasmid conjugation assay.

The whole genome from isolate G4584 was transformed into E. coli DH10B (Mem^−^ Kan^−^) (Invitrogen, USA) using electroporation, and carbapenem-resistant colonies were selected on CHROMagar KPC plates (CHROMagar, Paris, France). Transformation of *bla*_NDM-5_-containing plasmids was confirmed by PCR using NDM primers (NDM-Fwd, 5′-GTTTGGCGATCTGGTTTTC-3′; NDM-Rev, 5′-CGGAATGGCTCATCACGATC-3′). This NDM-5-containing E. coli DH10B/pG4584::NDM-5 (Mem^+^ Kan^−^) strain was used as a donor and cocultured with a recipient strain, E. coli JW2786-1 (Mem^−^ Kan^+^) (Coli Genetic Stock Center number 10181; Yale University, USA) on a Mueller-Hinton agar plate to promote conjugation. Transconjugants were selected on CHROMagar KPC plates containing 50 μg/ml of kanamycin. Simultaneously, donor and recipient strains were cultured separately and plated on CHROMagar KPC plates with 50 μg/ml of kanamycin as controls. Disk diffusion using meropenem, cefoxitin, cefepime, amoxicillin-clavulanic acid, and kanamycin antibiotic disks was performed for all the strains to determine the change in resistance. The zone diameter for each antibiotic was measured and interpreted according to Clinical and Laboratory Standards Institute (CLSI) guidelines (2017).

### Ethics statement.

This study was approved by the ethics committees of the Federal Capital Territory, University of Abuja Teaching Hospital, Gwagwalada, Nigeria, and the University of Nebraska Medical Center, Omaha, Nebraska, Institutional Review Board for the Community-Acquired Bacteremic Syndrome in Young Nigerian Children (CABSYNC).

### Accession number(s).

The genomes sequenced and analyzed in this study, as well as their associated metadata, are available at NCBI under BioProject no. PRJNA351846 with the following accession numbers: for G4582, NFXE00000000; for G4584, CP034129 to CP034135; for G4593, NFXD00000000; for G4601, NFXB00000000; for G4612, NFWY00000000; for G747, CP034136 to CP034140 and CP034339; and for G4704, NFWX00000000. In addition, AST results for strain G4584 for ceftazidime-avibactam, aztreonam, aztreonam-avibactam, and colistin are available under BioSample accession no. SAMN05960932.
