# Supplementary material for: Emergence of New Delhi Metallo-β-Lactamase (NDM-5) in Klebsiella quasipneumoniae from Neonates in a Nigerian Hospital
Source: mSphere. 2019 Mar 13;4(2):e00685-18. doi: 10.1128/mSphere.00685-18 (PMC6416368; doi:10.1128/mSphere.00685-18)
Supplement: TABLE S1 [file mSphere.00685-18-st001.pdf]

Table S1. *Klebsiella quasipneumoniae* subsp. s*imilipneumoniae* genomes analyzed as part of this study.

Biosample	NCBI Organism Name	Strain	MLST ST	Year Isolated	Country of Isolation	07A044^{T} ANI
SAMD00115699	*Klebsiella pneumoniae*	TUM1814	new	2003		98.75
SAMEA104305406	*Klebsiella pneumoniae*	4300STDY7045914	1031	2016		98.87
SAMEA2273559	*Klebsiella pneumoniae*	k482	new	2002	United Kingdom	98.99
SAMEA2273570	*Klebsiella pneumoniae*	k553	new	2003	United Kingdom	98.97
SAMEA2273581	*Klebsiella pneumoniae*	k644	384	2003	United Kingdom	98.97
SAMEA2273751	*Klebsiella pneumoniae*	k1821	1318	2008	United Kingdom	98.67
SAMEA2273767	*Klebsiella pneumoniae*	k2074	334	2009	United Kingdom	98.91
SAMEA23995918	*Klebsiella pneumoniae*	4300STDY6636950	1584	2016	Thailand	98.25
SAMEA24007168	*Klebsiella pneumoniae*	4300STDY6636965	1224	2016	Thailand	98.52
SAMEA24011668	*Klebsiella pneumoniae*	4300STDY6636971	1224	2016	Thailand	98.52
SAMEA24020668	*Klebsiella pneumoniae*	4300STDY6636983	new	2016	Thailand	98.48
SAMEA24026668	*Klebsiella pneumoniae*	4300STDY6636991	1584	2016	Thailand	98.3
SAMEA2609358	*Klebsiella quasipneumoniae subsp. similipneumoniae*	3189STDY5864734	new			98.74
SAMEA2609381	*Klebsiella quasipneumoniae subsp. similipneumoniae*	3189STDY5864741	1822	2010/2012	Pakistan	98.79
SAMEA2609441	*Klebsiella quasipneumoniae subsp. similipneumoniae*	3189STDY5864762	334	2010/2012	Pakistan	98.77
SAMEA2609448	*Klebsiella quasipneumoniae subsp. similipneumoniae*	3189STDY5864766	1822			98.78
SAMEA2609452	*Klebsiella quasipneumoniae subsp. similipneumoniae*	3189STDY5864768	new	2010/2012	Pakistan	98.78
SAMEA2609534	*Klebsiella quasipneumoniae subsp. similipneumoniae*	3189STDY5864907	334	2010/2012	Pakistan	98.7
SAMEA3138998	*Klebsiella quasipneumoniae subsp. similipneumoniae*	07A044	1215			100
SAMEA3207734	*Klebsiella pneumoniae ATCC 43816*	45T1-2A	new			98.83
SAMEA3403055	*Klebsiella pneumoniae*	NCTC13368	489	1994	USA	99.12
SAMEA3531564	*Klebsiella pneumoniae*	VRCO0013	138	2014	United Kingdom	99
SAMEA3531565	*Klebsiella pneumoniae*	VRCO0023	3011	2014	United Kingdom	98.93
SAMEA3531575	*Klebsiella pneumoniae*	VRCO0079	3013	2014	United Kingdom	99.13
SAMEA3531616	*Klebsiella pneumoniae*	VRES0419	477	2014	United Kingdom	98.91
SAMEA3531844	*Klebsiella pneumoniae*	PB439	477	2015	Thailand	98.64
SAMEA3531848	*Klebsiella pneumoniae*	PB463	1318	2015	Thailand	98.5
SAMEA3531864	*Klebsiella pneumoniae*	PB509	1318	2015	Thailand	98.52
SAMEA3531892	*Klebsiella pneumoniae*	W2-13-ERG3	2355	2015	Thailand	98.85
SAMEA3531893	*Klebsiella pneumoniae*	W2-13-ERG4	2355	2015	Thailand	98.85
SAMEA3940169	*Klebsiella quasipneumoniae subsp. similipneumoniae*	06-219	138	2006	Mexico	98.93
SAMEA4362626	*Klebsiella pneumoniae*	VRCO0334	740	2015	United Kingdom	98.92
SAMEA4362627	*Klebsiella pneumoniae*	VRCO0335	740	2015	United Kingdom	98.92
SAMEA4362628	*Klebsiella pneumoniae*	VRCO0336	740	2015	United Kingdom	98.92
SAMEA4362629	*Klebsiella pneumoniae*	VRCO0337	740	2015	United Kingdom	98.92
SAMEA4362630	*Klebsiella pneumoniae*	VRCO0338	740	2015	United Kingdom	98.92
SAMEA4362658	*Klebsiella pneumoniae*	VRCO0462	3019	2015	United Kingdom	98.95
SAMEA4362659	*Klebsiella pneumoniae*	VRCO0465	3019	2015	United Kingdom	98.94
SAMEA4362660	*Klebsiella pneumoniae*	VRCO0466	3019	2015	United Kingdom	98.94
SAMEA4362661	*Klebsiella pneumoniae*	VRCO0467	3019	2015	United Kingdom	98.95
SAMEA4362662	*Klebsiella pneumoniae*	VRCO0468	3019	2015	United Kingdom	98.95
SAMEA4362709	*Klebsiella pneumoniae*	VRCO0463	3019	2015	United Kingdom	98.95
SAMEA4364641	*Klebsiella pneumoniae*	4300STDY6470460	new	2016	Thailand	98.75
SAMEA4394726	*Klebsiella pneumoniae*	4300STDY6542356	new	2016	Thailand	98.87
SAMEA4394727	*Klebsiella pneumoniae*	4300STDY6542357	new	2016	Thailand	98.85
SAMEA4557830	*Klebsiella aerogenes*	NCTC9499	367			98.82
SAMN02045512	*Klebsiella pneumoniae subsp. pneumoniae*	12-3578	421	2012	China	98.7
SAMN02138584	*Klebsiella quasipneumoniae subsp. similipneumoniae*	MGH 44	1435			98.72
SAMN02471908	*Klebsiella pneumoniae 700603*	700603	489			99.11
SAMN03197266	*Klebsiella quasipneumoniae subsp. similipneumoniae*	1294_KPNE	new		USA	99.11
SAMN03197385	*Klebsiella quasipneumoniae subsp. similipneumoniae*	193_KOXY	new		USA	98.95

SAMN03197576	*Klebsiella quasipneumoniae subsp. similipneumoniae*	385_ECLO	367		USA	98.99
SAMN03274866	*Klebsiella pneumoniae*	1kgm	2045	2012	Malaysia	98.93
SAMN03280408	*Klebsiella pneumoniae*	MGH123	new	2014		98.7
SAMN03280418	*Klebsiella pneumoniae*	BIDMC95	1535	2014		98.83
SAMN03945398	*Klebsiella variicola*	HKUOPLA	new	2013	Hong Kong	98.98
SAMN03955386	*Klebsiella pneumoniae subsp. pneumoniae*	HKUOPLC	new	2013	Hong Kong	98.98
SAMN03996259	*Klebsiella quasipneumoniae*	ATCC 700603	489	1994	USA	99.12
SAMN04014921	*Klebsiella pneumoniae*	AR_0080	1224			98.58
SAMN04215234	*Klebsiella pneumoniae*	II EMP3	new	2013	Indonesia	98.74
SAMN04382091	*Klebsiella quasipneumoniae*	ATCC 700603	489	1994	USA	99.12
SAMN04436401	*Klebsiella quasipneumoniae*	HKUOPA4	new	2013	China	98.98
SAMN04555532	*Klebsiella quasipneumoniae subsp. similipneumoniae*	KP-Z4175	new	2013	USA	98.77
SAMN05170810	*Klebsiella quasipneumoniae subsp. similipneumoniae*	SKLX2736	138	2011	China	98.88
SAMN05170868	*Klebsiella quasipneumoniae subsp. similipneumoniae*	SKLX2781	1031	2012	China	98.93
SAMN05178559	*Klebsiella quasipneumoniae subsp. similipneumoniae*	MB373	new	2013	Pakistan	98.76
SAMN05225354	*Klebsiella quasipneumoniae*	B8095	new	2015	India	98.63
SAMN05505619	*Klebsiella quasipneumoniae*	CCBH16302	138	2014	Brazil	98.64
SAMN05960874	*Klebsiella quasipneumoniae subsp. similipneumoniae*	PO416	1031	2009	Nigeria	98.93
SAMN05960909	*Klebsiella quasipneumoniae subsp. similipneumoniae*	CAB1577	1224	2013	Nigeria	98.47
SAMN05960914	*Klebsiella quasipneumoniae subsp. similipneumoniae*	G747	476	2013	Nigeria	98.65
SAMN05960931	*Klebsiella quasipneumoniae subsp. similipneumoniae*	G4582	476	2016	Nigeria	98.59
SAMN05960932	*Klebsiella quasipneumoniae*	G4584	476	2016	Nigeria	98.59
SAMN05960934	*Klebsiella quasipneumoniae subsp. similipneumoniae*	G4593	476	2016	Nigeria	98.59
SAMN05960936	*Klebsiella quasipneumoniae subsp. similipneumoniae*	G4601	476	2016	Nigeria	98.59
SAMN05960939	*Klebsiella quasipneumoniae subsp. similipneumoniae*	G4612	476	2016	Nigeria	98.6
SAMN05960940	*Klebsiella quasipneumoniae subsp. similipneumoniae*	G4704	1031	2016	Nigeria	98.93
SAMN05960967	*Klebsiella quasipneumoniae subsp. similipneumoniae*	MM_2867	1584	2016	Nigeria	98.68
SAMN05960968	*Klebsiella quasipneumoniae subsp. similipneumoniae*	UNMC_7493	1584	2013	Nigeria	98.68
SAMN06629584	*Klebsiella quasipneumoniae*	COL-Kpn7	1191	2003	Colombia	99.02
SAMN06629588	*Klebsiella quasipneumoniae*	COL-Kpn11	1584	2003	Colombia	98.54
SAMN06629596	*Klebsiella quasipneumoniae*	COL-Kpn19	339	2006	Colombia	98.71
SAMN06629621	*Klebsiella quasipneumoniae*	COL-Kpn44	339	2006	Colombia	98.7
SAMN06629693	*Klebsiella quasipneumoniae*	COL-Kpn116	1584	2003	Colombia	98.58
SAMN06629701	*Klebsiella quasipneumoniae*	COL-Kpn124	138	2002	Colombia	99.01
SAMN06629704	*Klebsiella quasipneumoniae*	COL-Kpn127	477	2002	Colombia	98.71
SAMN06767172	*Klebsiella quasipneumoniae*	KPC142	new	2011	Brazil	98.64
SAMN06885982	*Klebsiella quasipneumoniae*	MGF005	1031	2012	Malaysia	98.93
SAMN06886016	*Klebsiella quasipneumoniae*	MGF008	1031	2015	Malaysia	98.93
SAMN07167861	*Klebsiella quasipneumoniae*	DL5.4	new	2011	India	99.11
SAMN07175655	*Klebsiella quasipneumoniae*	AUH-KIMP304	2720	2014	Lebanon	99.01
SAMN07176438	*Klebsiella quasipneumoniae*	AUH-KIMP307	2720	2014	Lebanon	99.03
SAMN07450550	*Klebsiella quasipneumoniae subsp. similipneumoniae*	KPCRETH06	1770	2016	Thailand	98.57
SAMN07450604	*Klebsiella quasipneumoniae subsp. similipneumoniae*	KPCTRSRTH05	421	2016	Thailand	98.81
SAMN07450605	*Klebsiella quasipneumoniae subsp. similipneumoniae*	KPCTRSRTH04	1318	2016	Thailand	98.58
SAMN07450606	*Klebsiella quasipneumoniae subsp. similipneumoniae*	KPCTRSRTH03	3387	2016	Thailand	98.64
SAMN07450695	*Klebsiella quasipneumoniae subsp. similipneumoniae*	KPPSTH03	new	2016	Thailand	98.73
SAMN07609112	*Klebsiella quasipneumoniae subsp. similipneumoniae*	SCKQ020050	1859	2016	China	98.59
SAMN07692774	*Klebsiella quasipneumoniae*	46	1887	2015	Ghana	98.79
SAMN08623804	*Klebsiella quasipneumoniae*	CRE30	384	2015	USA	98.78
SAMN08885787	*Klebsiella pneumoniae*	S009	new	2013	USA	98.86
SAMN09245763	*Klebsiella quasipneumoniae subsp. similipneumoniae*	ATCC 700603	489	1994	USA	99.12
