# Supplementary material for: Emergence of New Delhi Metallo-β-Lactamase (NDM-5) in Klebsiella quasipneumoniae from Neonates in a Nigerian Hospital
Source: mSphere. 2019 Mar 13;4(2):e00685-18. doi: 10.1128/mSphere.00685-18 (PMC6416368; doi:10.1128/mSphere.00685-18)
Supplement: TABLE S2 [file mSphere.00685-18-st002.pdf]

Table S2. *In Silico* predicted antimicrobial resistance genes.

Gene	G747	G4582	G4584	G4593	G4601	G4612	G4704
AAC(3)-IIb	77.15%	76.12%	76.12%	76.12%	76.12%	76.12%	77.15%
AAC(6')-Ib-cr		100%	100%	100%	100%	100%	
acrB	91.04%	91.13%	91.13%	91.13%	91.13%	91.13%	91.04%
adeF	41.95%	41.95%	41.95%	41.95%	41.95%	41.95%	41.95%
adeF	61.15%	61.05%	61.05%	61.05%	61.05%	61.05%	61.15%
APH(3'')-Ib	99.63%	99.63%	99.63%	99.63%	99.63%	99.63%	99.63%
APH(6)-Id	99.64%	99.64%	99.64%	99.64%	99.64%	99.64%	99.64%
baeR	92.08%	92.08%	92.08%	92.08%	92.08%	92.08%	92.08%
BRP(MBL)		100%	100%	100%	100%	100%	
catII	91.08%						91.08%
CRP	99.05%	99.05%	99.05%	99.05%	99.05%	99.05%	99.05%
CTX-M-15		100%	100%	100%	100%	100%	
CTX-M-14	100%						100%
dfrA5		89.81%	90.45%	89.81%	89.81%		
dfrA14						99.34%	
dfrA15	91.72%						91.72%
emrB	94.02%	93.82%	93.82%	93.82%	93.82%	93.82%	94.02%
emrR	92.57%	92.57%	92.57%	92.57%	92.57%	92.57%	92.57%
Escherichia coli EF-Tu with mutation			97.97%*				
Escherichia coli marR with mutation	83.33%	83.33%	83.33%	83.33%	83.33%	83.33%	83.33%
Escherichia coli mdfA	85.37%	85.37%	85.37%	85.37%	85.37%	85.37%	85.37%
Escherichia coli soxS with mutation	89.72%	89.72%	89.72%	89.72%	89.72%	89.72%	89.72%
Escherichia coli uhpT with mutation	95.25%	94.82%	94.82%	94.82%	94.82%	94.82%	95.25%
fosA2	70.59%	71.32%	71.32%	71.32%	71.32%	71.32%	70.59%
Haemophilus influenzae PBP3	52.2%	52.2%	52.2%	52.2%	52.2%	52.2%	52.2%
Klebsiella pneumoniae acrR with mutation	98.61%	98.61%	98.61%	98.61%	98.61%	98.61%	98.61%
Klebsiella pneumoniae ompK37	95.72%	95.99%	95.99%	95.99%	95.99%	95.99%	95.72%
marA	92.74%	92.74%	92.74%	92.74%	92.74%	92.74%	92.74%
mdtB	90.19%	90.19%	90.19%	90.19%	90.19%	90.19%	90.19%
mdtC	91.51%	91.51%	91.51%	91.51%	91.51%	91.51%	91.51%
msbA	92.61%	92.61%	92.61%	92.61%	92.61%	92.61%	92.61%
NDM-5		100%	100%	100%	100%	100%	
OKP-B-1	100%						100%
OKP-B-6		100%	100%	100%	100%	100%	
oqxA	96.93%	97.44%	97.44%	97.44%	97.44%	97.44%	96.93%
OXA-1	100%	100%	100%	100%	100%	100%	
patA	94.12%	93.9%	93.9%	93.9%	93.9%	93.9%	94.12%
pmrF	83.69%	83.69%	83.69%	83.69%	83.69%	83.69%	83.69%
qnrB1		100%	100%	100%	100%	100%	
qnrS1	100%						100%
sul1	100%						100%
sul2	100%	100%	100%	100%	100%	100%	100%
TEM-1	100%	100%	100%	100%	100%	100%	100%
tet(59)	67.62%						67.62%

tet(C)	79.39%	79.39%	79.39%	79.39%	79.39%	79.39%	79.39%
vgaC		94.59%	94.59%	94.59%	94.59%	94.59%	
